# Comparison of spasmolytic regimen for prevention of radial artery spasm during the distal radial approach: A single-center, randomized study

**DOI:** 10.3389/fcvm.2023.1007147

**Published:** 2023-03-01

**Authors:** Oh-Hyun Lee, Ji Woong Roh, Yongcheol Kim, Nak-Hoon Son, Jay Yi Cho, Daesek Jang, Eui Im, Deok-Kyu Cho, Donghoon Choi

**Affiliations:** ^1^Division of Cardiology, Department of Internal Medicine, Yonsei University College of Medicine and Cardiovascular Center, Yongin Severance Hospital, Yongin, South Korea; ^2^Department of Statistics, Keimyung University, Daegu, South Korea; ^3^Division of Cardiology, Severance Cardiovascular Hospital, Yonsei University College of Medicine, Seoul, South Korea

**Keywords:** radial artery spasm, vasodilators, coronary angiography, distal radial approach, percutaneous coronary intervention

## Abstract

**Background:**

The distal radial approach (DRA) for coronary catheterization is increasingly being used worldwide yet the optimal medication regimen to prevent radial artery spasm (RAS), an important factor for the success of the procedure, remains unclear. The aim of this study is to examine the effectiveness of medication for preventing RAS via the DRA.

**Methods:**

This was a prospective, comparative randomized study including 400 patients who underwent coronary catheterization via DRA in single center by three experienced DRA operators. Patients were randomized to either nitroglycerin (NTG) injection (*N* = 200) or NTG plus verapamil (*N* = 200) to compare the effectiveness and safety of these regimens.

**Results:**

There were no differences between the groups in the changes in radial artery diameter at most spastic area (0.34 ± 0.20 in the NTG group, 0.35 ± 0.20 in the NTG plus verapamil group; *P* = 0.73). There was no difference between the groups in the ratio of patients without arm pain during the procedure (95.0% in the NTG group, 93.5% in the NTG plus verapamil group; *P* = 0.67). However, there was a greater reduction in diastolic blood pressure in the NTG plus verapamil group (–8.3 ± 7.9 mmHg) than in the NTG group (–6.6 ± 7.6 mmHg) (*P* = 0.03).

**Conclusion:**

Intra-arterial injection of NTG as a single agent is effective and safe in the prevention of RAS during coronary catheterization via the DRA compared with a cocktail regimen of NTG plus verapamil.

**Clinical trial registration:**

https://cris.nih.go.kr, identifier KCT0005177.

## Highlights

- Compared with intra-arterial injection of nitroglycerin plus verapamil, administration of nitroglycerin as a single agent is effective and safe in preventing radial artery spasm during coronary catheterization via the distal radial approach. A 3,000 unit of heparin was administered to all patients via an intra-arterial sheath to prevent radial artery occlusion on top of either cocktail or NTG solution.
- The combination of nitroglycerin plus verapamil is associated with a transiently decrease in diastolic blood pressure compared with usage of only nitroglycerin without adverse symptom.

## Introduction

Current guidelines recommend the transradial approach as the standard approach for coronary angiography (CAG) and percutaneous coronary intervention (PCI) (1, 2). Radial artery spasm (RAS) is one of the most frequently encountered complications of transradial coronary artery procedures. RAS occurs in up to 51.3% of cases (3) and has been identified as a major cause of access failure. It can result in considerable pain and may lead to failure of the procedure (4). Furthermore, RAS is a risk factor for post-procedural radial artery occlusion (RAO) (5, 6).

The prevention of RAS is achieved by the spasmolytic agents including intra-arterial vasodilators such as verapamil or nitroglycerin (NTG), with or without heparin (7). The 2018 Scientific Statement from the American Heart Association on the best transradial approach practices endorses the use of intra-arterial NTG and/or calcium channel blockers after sheath insertion (8). However, verapamil may have side effects and is contraindicated in some patients such as those with severe hypotension, second- or third-degree atrioventricular block, and severe congestive heart failure (9), thus the inclusion of verapamil in the spasmolytic agents varies from hospital to hospital.

Recently, the distal radial approach (DRA) for CAG or PCI, has emerged as an alternative to the proximal radial approach (PRA) with shorter hemostasis time and fewer access-site complications (10–12). Furthermore, the DRA prevents RAO after the procedure (13, 14) although the distal radial artery has a smaller diameter compared to the proximal radial artery. Despite growing interest in the DRA, there is a lack of data regarding the effects of spasmolytic agents to prevent RAS in patients undergoing CAG or PCI via the DRA. The present study compared the spasmolytic effects of NTG with those of a cocktail using NTG and verapamil before the coronary procedure via DRA.

## Materials and methods

### Study population

The V-SPA (comparison of spasmolytic regimen for the preVention of radial artery SPasm during coronary angiography via the distal radial Approach) was an investigator-initiated, single-center, prospective randomized single (patient) blinded study, conducted in Yongin Severance Hospital, South Korea. Between June 2020 and July 2021, patients with a well palpable distal radial artery, who underwent a coronary angiography via the DRA, were enrolled in this study. The following exclusion criteria applied: (1) acute coronary syndrome, (2) hypotension (systolic blood pressure ≤ 90 mmHg), (3) marked bradycardia (pulse rate ≤50 beats per min), (4) 2nd or 3rd atrioventricular block, (5) severe left ventricular systolic dysfunction (left ventricular ejection fraction ≤30%) with or without acute pulmonary edema, (6) pregnancy, (7) use of an oral vasodilator such as nitrate, nicorandil, verapamil or diltiazem within 1 week of the coronary angiography. This study protocol was approved by the Institutional Review Board of Yongin Severance Hospital (approval number: 9-2020-0047), and all participants provided written informed consent before participating in the study. The study protocol was registered on the CRIS (Clinical Research Information Service) and adhered to the ethical guidelines of the Declaration of Helsinki.

### DRA

Coronary catheterization was performed by three interventional cardiologists with extensive experience in the DRA, defined as operators who perform at least 50% of all PCI procedures via the DRA. Sedatives or opioids were not administered during the entire procedure. After local subcutaneous anesthesia with 2% lidocaine hydrochloride 1cc at the anatomical snuffbox area, distal radial arterial puncture was performed using a 20-gauge 2-piece needle or a 21-gauge open needle. The needle was directed to the point of the strongest pulse within or outside the anatomical snuffbox area. Then, a 0.018-inch hair wire was placed in the radial artery, followed by advancement of the 4-, 5- or 6-Fr, 11 cm length hydrophilic thin-wall sheath (Prelude IDEAL™, Merit Medical, South Jordan, UT, USA) on the guidewire in all patients. A 3,000 unit of heparin was administered to all patients via an intra-arterial sheath.

### Randomization and study protocol

After successful cannulation, radial artery angiography was acquired using an intra-arterial sheath. Patients were then randomly assigned (1:1) by block randomization to either receive only 200 μg of NTG (NTG group) or 200 μg of NTG plus 2 mg of verapamil (Cocktail group), which were diluted in 10 ml of normal saline. The allocation sequence was determined by a computed-generated random number table, with random block sizes of 4, 6, or 8 provided by an independent statistician.

Non-invasive brachial systolic blood pressure was measured before the procedure and after injection of spasmolytic agents. The maximum change was checked by repeatedly measuring the blood pressure every minute for 5 min after injection of the spasmolytic agents.

Repeated radial artery angiography was performed after the injection of spasmolytic agents. The radial artery diameter measurement was calculated twice, before and after the injection of the spasmolytic regimens, by quantitative computed angiography (QCA) using CASS workstation 7.4 (Pie Medical Imaging, Maastricht, Netherlands). All of the radial artery QCA images were analyzed by two independent observers with more than 5 years of experience in cardiac catheterization. All diameters were determined as the average of the diameter values obtained independently. The segments for analysis were the most spastic area and segments of the proximal and distal 2 cm of the corresponding area as the proximal and distal reference diameter, respectively. The reference diameter was determined by the mean of the proximal reference and the distal reference diameter of 2 cm length. The same area was measured using the same method after injection of the spasmolytic agents (Supplementary Figure 1).

Following radial artery angiography, CAG and PCI were performed according to the standard technique. Additional unfractionated heparin (50–70 U/kg) was administered during the procedure to maintain the activated clotting time at 250–300 s. For CAG, left and right Judkins catheters were used as the first choice. The catheters were advanced and exchanged on a 0.035-inch guide wire. If PCI was indicated, it was performed in most cases immediately after the diagnostic CAG. When *ad hoc* angioplasty was indicated, the 4- or 5-Fr sheath was exchanged by a 6, 7-Fr sheath or sheathless guiding catheter, as appropriate. The treatment strategy for coronary lesion including guiding catheter selection, balloon angioplasty or stenting, use of intravascular modalities, and use of an addition device (e.g., microcatheter and guide extension catheter) were determined at the operator’s discretion. After the completion of the procedure, the radial sheath was removed and a compressive cohesive elastic bandage with a 4 × 4- inch sterile gauze dressing for hemostasis was applied for 3 h in all patients and clinical RAS was evaluated by the independent investigator.

### Definition and study endpoints

The primary endpoint was the mean change of radial artery diameter at the most spastic area on the angiography from baseline after spasmolytic agent administration under both regimens. The secondary endpoints were clinically and hemodynamically adverse events associated with spasmolytic agent injection, and clinical RAS.

Clinical RAS was assessed according to symptoms and severity and classification was as follows: Grade 0, absence of arm pain or discomfort during and immediately after the procedure; Grade 1, mild spasm, minimal local pain and discomfort during catheter movement and/or immediate post procedure period; Grade 2, moderate spasm, significant local pain and discomfort during catheter movement (although completion of the procedure was possible) and/or in the immediate post procedure period; Grade 3, severe spasm, severe local pain and discomfort during catheter movement compelling the operator to stop the procedure and cross-over to the other route; Grade 4, very severe spasm, severe local pain and discomfort associated with catheter trapping (15). The maximal change in arterial blood pressure (BP) and heart rate (HR) was assessed within 5 min of injection of the spasmolytic agents, and compared to the baseline BP and HR prior to the angiography. In addition, symptoms associated with the medications administered were also evaluated including chest discomfort, dizziness and dyspnea.

### Statistical analyses

Normally distributed continuous variables are expressed as means ± standard deviations (SD) and were compared using the Student’s and paired *t* tests. Non-normally distributed continuous variables are reported as medians and interquartile ranges (IQR) and were compared using the Mann–Whitney *U* test. Categorical variables are presented as percentile values and were compared with the Chi-square or Fisher’s exact tests as appropriate. Inter-observer and intra-observer variation were assessed by the Tukey method and the difference between two measurements was regarded as not different if the *P* value was more than 0.05, meaning that the difference is located within 95% confidence interval. All statistical analyses were performed with SPSS statistical software (SPSS version 25.0 for Windows; IBM Corp., Armonk, NY, USA).

## Results

### Baseline, angiographic and procedural characteristics

**FIGURE 1:**
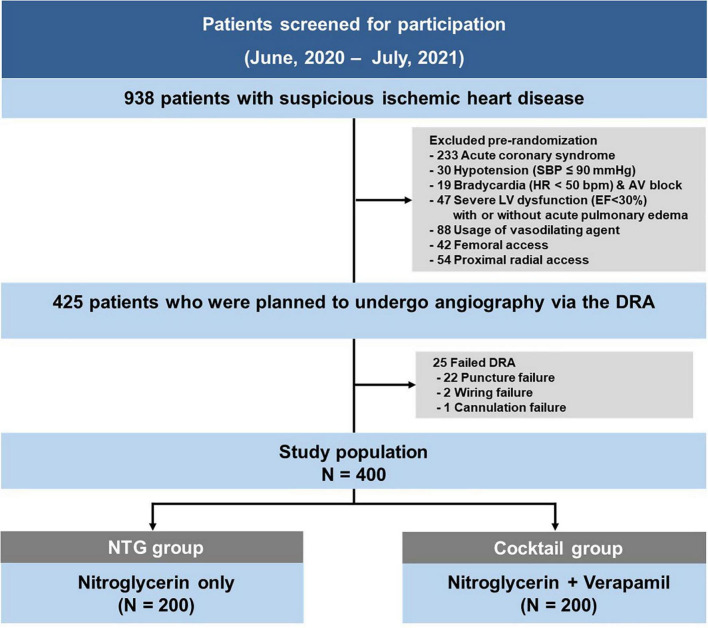
Study flowchart.

Between June 2020 and July 2021, total 938 coronary procedures were performed. Following the exclusion of 513 patients according to the pre-defined criteria, the DRA was attempted in 425 patients during the study period. The success rate of the DRA was 94.1% (400/425). Among the 25 cases of failed DRA, puncture failed in 22 patients, while wiring or sheath cannulation failed after successful puncture in 2 and 1 cases, respectively. The study included 400 patients (average age 65.2 ± 11.5 years; 70.8% men), randomized into two groups (200 in the NTG group, and 200 in the cocktail group) (Figure 1).

Baseline population characteristics by randomization group are presented in Table 1. There were no differences in baseline characteristics and medications used between the two groups. Angiographic and procedural characteristics were summarized in Table 2. Puncture time and number of attempt to puncture were comparable between the two groups. A total of 96% of patients underwent CAG through the left DRA (NTG group 96.5% vs. Cocktail group 95.5%, *P* = 0.61). Cross-over occurred in the NTG group with three patients due to tortuosity of the right subclavian artery (alpha loop) in two patients and cross-over after CAG for complex PCI in one patient. Final sheath size, catheter size, and catheter number were comparable between the two groups. Procedural time, contrast volume, and hemostasis time were also similar between the two groups.

**TABLE 1:** Baseline characteristics.

Characteristics	NTG group (*n* = 200)	Cocktail group (*n* = 200)	*P*-value
Age, years	65.6 ± 12.0	64.7 ± 11.0	0.44
Male gender	145 (72.5)	138 (69.0)	0.44
Height, cm	163.3 ± 9.5	163.1 ± 9.1	0.81
Weight, kg	68.1 ± 11.9	68.2 ± 11.0	0.93
Body mass index, kg/m^2^	25.4 ± 3.2	25.6 ± 3.2	0.61
Hypertension	129 (64.5)	127 (63.5)	0.84
Diabetes mellitus	76 (38.0)	87 (43.5)	0.26
Dyslipidemia	166 (83.0)	171 (85.5)	0.49
Current smoking	34 (17.0)	32 (16.0)	0.79
Prior percutaneous coronary intervention	48 (24.0)	38 (19.0)	0.22
Prior myocardial infarction	25 (12.5)	27 (13.5)	0.77
Prior cerebrovascular accident	17 (8.5)	13 (6.5)	0.45
Congestive heart failure	18 (9.0)	13 (6.5)	0.35
Atrial fibrillation	7 (3.5)	12 (6.0)	0.24
Chronic kidney disease, ≥stage 3	32 (16.0)	29 (14.5)	0.68
Dialysis	3 (1.5)	4 (2.0)	1.00
LVEF, %	57.5 ± 9.5	58.5 ± 8.2	0.29
**Laboratory findings**			
Total cholesterol, mg/dL	154.8 ± 43.0	155.9 ± 42.0	0.80
Triglyceride, mg/dL	140.8 ± 74.6	148.7 ± 101.6	0.40
HDL-cholesterol, mg/dL	49.4 ± 11.5	50.3 ± 12.9	0.52
LDL-cholesterol, mg/dL	95.7 ± 40.8	94.5 ± 36.4	0.79
Creatinine, mg/dL	0.96 ± 0.63	1.05 ± 1.02	0.32
Hemoglobin, g/dL	13.9 ± 1.6	14.1 ± 1.7	0.14
Platelet count, 10^3^/μL	227.0 ± 70.4	224.1 ± 59.4	0.67
CRP, mg/L	7.5 ± 20.3	7.8 ± 33.3	0.92
**Discharge medication**			
Aspirin	167 (83.9)	159 (79.9)	0.30
P2Y12 inhibitor	159 (79.5)	156 (78.0)	0.60
Clopidogrel	151 (95.0)	150 (96.2)	
Ticagrelor	8 (5.0)	5 (3.2)	
Prasugrel	0	1 (0.6)	
Oral anticoagulation	11 (5.5)	13 (6.5)	0.67
ACEi or ARB	99 (49.5)	88 (44.0)	0.27
Beta-blocker	55 (27.5)	64 (32.0)	0.33
Calcium channel blocker	55 (27.5)	53 (26.5)	0.82
Statin	165 (82.5)	164 (82.0)	0.90

Data are presented as the mean ± SD or number (%). NSTEMI, non-ST-segment elevation myocardial infarction; STEMI, ST-segment elevation myocardial infarction; LVEF, left ventricular ejection fraction; HDL high density lipoprotein; LDL, low density lipoprotein; CRP, C-reactive protein, ACEi, angiotensin converting enzyme inhibitor; ARB, angiotensin receptor blocker.

**TABLE 2:** Angiographic and procedural characteristics.

Characteristics	NTG group (*n* = 200)	Cocktail group (*n* = 200)	*P*-value
Puncture time, sec	150.6 ± 125.3	149.6 ± 119.9	0.94
Attempts to puncture, frequency	1.1 ± 0.4	1.2 ± 0.5	0.83
Puncture success with 1 attempt	177 (88.5)	180 (90.0)	0.63
Left distal radial access	193 (96.5)	191 (95.5)	0.61
Crossover	3 (1.5)	0 (0)	–
Success rate of CAG	198 (99.0)	200 (100)	0.50
Success rate of PCI	53 (100)	44 (100)	–
Physiologic assessment (FFR)	18 (9.0)	16 (8.0)	0.72
Initial sheath size during the spasmolytic regimens injection			0.47
4 Fr	15 (7.5)	22 (11.0)	
5 Fr	166 (83.0)	161 (80.5)	
6 Fr	19 (9.5)	17 (8.5)	
Final sheath size			0.69
4 Fr	14 (7.0)	17 (8.5)	
5 Fr	136 (68.0)	139 (69.5)	
6 Fr	46 (23.0)	38 (19.0)	
7 Fr	4 (2.0)	6 (3.0)	
Pain scale during sheath insertion, NRS	3.5 ± 2.2	3.4 ± 2.3	0.45
Catheter size			0.94
4 Fr	14 (7.0)	16 (8.0)	
5 Fr	144 (72.0)	142 (71.0)	
6 Fr	32 (16.0)	30 (15.0)	
7 Fr	5 (2.5)	8 (4.0)	
6.5 Fr Sheathless	3 (1.5)	3 (1.5)	
7.5 Fr Sheathless	2 (1.0)	1 (0.5)	
Catheter number	2.3 ± 0.7	2.3 ± 0.6	0.64
Procedure time, min	30.4 ± 26.3	28.6 ± 24.1	0.49
Contrast volume, cc	124.4 ± 86.1	124.1 ± 82.3	0.97
Hemostasis time, min	173.4 ± 81.1	174.1 ± 78.8	0.93

Data are presented as the mean ± SD, median or number (%). NRS, neumerical rating scale; FFR, fractional flow reserve; PCI, percutaneous coronary intervention; ACT, activated clotting time.

### Comparison of efficacy outcomes

**FIGURE 2:**
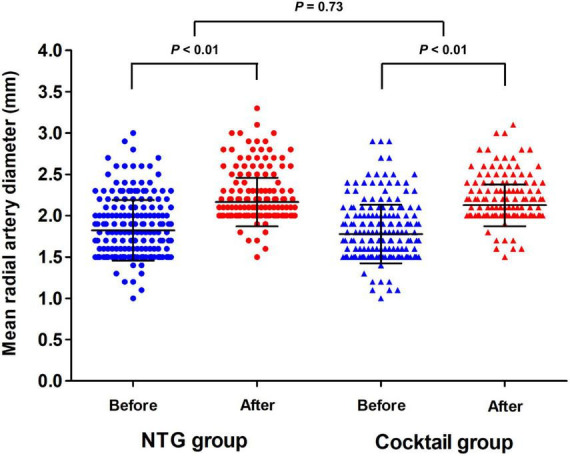
Comparison of mean radial artery diameter in the most spastic area between the NTG and cocktail group.

Changes in the mean diameter of the radial artery in both groups are summarized in Table 3, Figure 2, and Supplementary Table 1). The mean diameter at the most spastic portion of the radial artery before the injection of the spasmolytic agents were similar between the two groups (1.83 ± 0.35 vs. 1.79 ± 0.33 mm, *P* = 0.23). After administration of the spasmolytic agents, the mean diameter of the vessel increased significantly in both groups (Supplementary Table 1), although there was no difference between the groups in the change in mean diameter. These tendencies also observed in diameter at the reference areas between two groups (Table 3).

**TABLE 3:** Efficacy outcomes.

Variables	NTG group (*n* = 200)	Cocktail group (*n* = 200)	*P*-value
**Radial artery at the most spastic area, mm**			
Pre-radial artery diameter	1.83 ± 0.35	1.79 ± 0.33	0.23
Post-radial artery diameter	2.18 ± 0.28	2.14 ± 0.23	0.18
Δ diameter	0.34 ± 0.20	0.35 ± 0.20	0.73
**Reference diameter of radial artery, mm**			
Pre-radial artery diameter	2.66 ± 0.16	2.65 ± 0.13	0.42
Post-radial artery diameter	2.90 ± 0.21	2.89 ± 0.15	0.46
Δ diameter	0.25 ± 0.12	0.24 ± 0.10	0.87
Clinical RAS			0.81
Grade 0	190 (95.0)	187 (93.5)	0.67
Grade 1	7 (3.5)	9 (4.5)	0.80
Grade 2	3 (1.5)	4 (2.0)	0.99
Grade 3	0	0	–
Grade 4	0	0	–
Treatment for RAS	8 (4.0)	8 (4.0)	1.00

Data are presented as the mean ± SD, median or number (%). RAS, radial artery spasm.

In terms of the clinical spasm, a total of 94.3% patients did not complain of any arm discomfort or pain (RAS grade 0) during the CAG or PCI (95% in NTG group vs. 93.5% in the Cocktail group, *P* = 0.67) and there were no differences between the two groups in the incidence of spasm and the distribution of spasm severity (Table 3). Clinically significant RAS (RAS ≥ 2) occurred in 3 and 4 patients in the NTG group, and cocktail group, respectively (Supplementary Table 2). Eight patients (4.0%) in both groups required additional vasodilator injection during the procedure due to radial artery spasm, although no patients needed cross-over to another vascular access due to spasm.

### Safety outcomes

**FIGURE 3:**
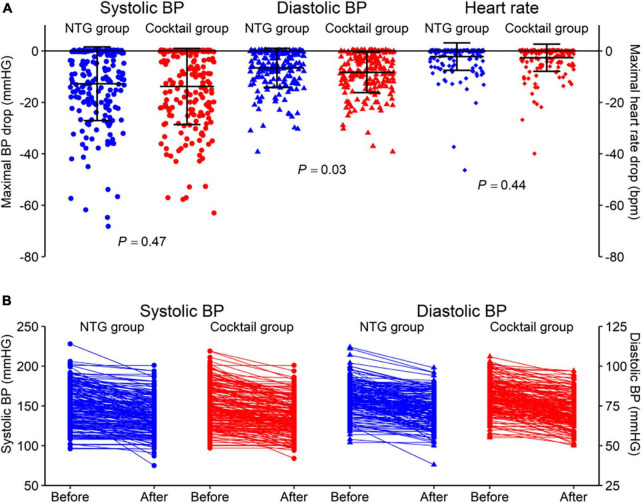
**(A)** Comparison of changes in vital signs including systolic and diastolic blood pressure and heart rate between the NTG and cocktail group. **(B)** Changes of systolic and diastolic blood pressure in NTG and cocktail group.

Vital signs and adverse effects associated with injection of spasmolytic agents are listed in Table 4 and Figure 3. There was no difference in blood pressure and heart rate changes in both groups before and after injection of the spasmolytic agents. However, after injection of the spasmolytic agents, diastolic blood pressure transiently decreased more significantly in the cocktail group than in the NTG group (–6.6 ± 7.6 mmHg in NTG group vs. −8.3 ± 7.9 mmHg in Cocktail group, *P* = 0.03). There were no reports of chest discomfort, dyspnea or dizziness after injection of the spasmolytic agents.

**TABLE 4:** Safety outcomes.

Variables	NTG group (*n* = 200)	Cocktail group (*n* = 200)	*P*-value
Systolic blood pressure, mmHg	149.7 ± 27.0	149.2 ± 26.4	0.88
Diastolic blood pressure, mmHg	79.0 ± 10.9	80.8 ± 10.2	0.10
Heart rate, bpm	70.0 ± 12.9	70.2 ± 11.5	0.89
Δ systolic blood pressure, mmHg	−12.8 ± 14.3	−13.8 ± 14.8	0.47
Δ diastolic blood pressure, mmHg	−6.6 ± 7.6	−8.3 ± 7.9	0.03
Δ heart rate, bpm	−2.2 ± 5.3	−2.6 ± 5.3	0.44
**Adverse effect**			
Chest discomfort	0	0	–
Dyspnea	0	0	–
Dizziness	0	0	–
**Access-site complication**			
Local hematoma (<5 cm)	2 (1.0)	3 (1.5)	1.00
Local hematoma (≥5 cm)	0	0	–
Radial artery occlusion	0	0	–
Numbness	0	0	–
Radial artery dissection	0	0	–
Radial artery rupture	0	0	–
Arteriovenous fistula	0	0	–
Pseudoaneurysm	0	0	–

Data are presented as the mean ± SD, median or number (%). bpm, beats per minute.

## Discussion

**FIGURE 4:**
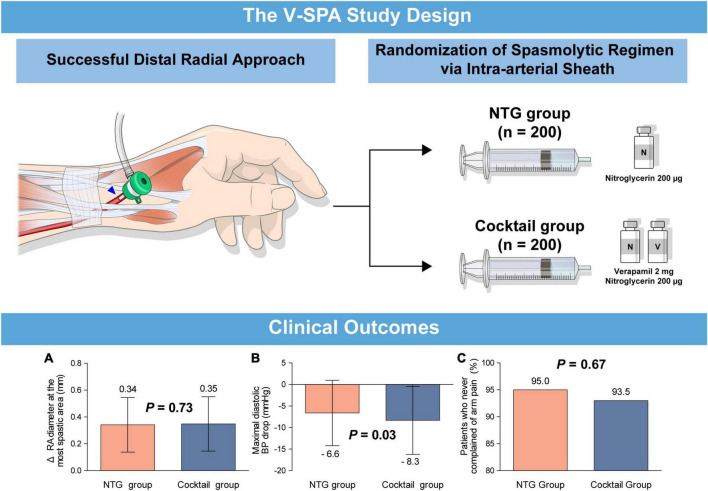
(Visual abstract). Comparison of spasmolytic regimen (only NTG versus NTG plus verapamil) for prevention of radial artery spasm during the distal radial approach. A prospective, randomized, single-center trial, the V-SPA study, demonstrated that intra-arterial injection of NTG as a single agent is effective and safe in the prevention of RAS during coronary catheterization via the DRA compared with a cocktail regimen of NTG plus verapamil. **(A)** Changes of radial artery diameter at the most spastic area, **(B)** maximal diastolic blood pressure drop, **(C)** patients who never complained of arm pain. DRA, distal radial approach; NTG, nitroglycerin; RAS, radial artery spasm; V-SPA, comparison of spasmolytic regimen for the preVention of radial artery SPasm during coronary angiography via the distal radial approach.

The principal findings of the current study were as follows (Figure 4): in patients undergoing coronary catheterization via the DRA. First, injection of NTG only via intra-arterial sheath is effective and safe for the prevention of RAS demonstrating radial artery dilation and no arm pain in 95% of patients. This result is comparable to that observed in the group treated with the cocktail solution of NTG and verapamil. Second, the use of NTG plus verapamil via intra-arterial sheath is associated with a transient decrease in diastolic blood pressure with no change in the heart rate compared with injection of NTG only. To the best of our knowledge, this is the first study to investigate the effects of spasmolytic agents used during cardiac catheterization via the DRA.

RAS during elective or urgent PCI results in severe limitations for PRA specifically, in the ability to manipulate either the wire or catheter, together with considerable pain, increased procedure times, exposure to radiation, and increased costs (16). Therefore, many studies examining the spasmolytic solution for the prevention of RAS were conducted when performing PRA (Supplementary Table 3). Regarding the spasmolytic agents used to prevent RAS with the DRA, it is postulated that most institutions use the same regimen for PRA when performing DRA because there are no recommendations on the DRA in the current guidelines. However, the DRA is increasingly being used worldwide since Kiemeneij firstly introduced this approach in coronary procedures in 2017 (17). Two recently published randomized trials comparing the DRA with PRA, used different drug regimens to prevent arterial spasm: NTG 200 μg versus verapamil 2.5 mg plus NTG 200 μg (13, 14).

In the current study, we demonstrated that the injection of only NTG via intra-arterial sheath had a similar vasodilator effect, confirmed by quantitative analysis using QCA when compared with the combination of NTG and verapamil for DRA. In addition, the incidence of RAS was not high in both groups (NTG group vs. Cocktail group; Any RAS, 5.0% vs. 6.5%, *P* = 0.52; RAS ≥ Grade 2, 1.5% vs. 2.0%, *P* = 0.99). These are relatively low rates of spasm when compared with the results of previous studies on the PRA (7). Regarding the lower prevalence of RAS in the current study population, this could be explained by the following. First, highly experienced DRA operators conducted this dedicated DRA study. The DRA success rate (96%) of this study is significantly higher than that of a previous randomized study (13). In addition, the proportion of patients who were successfully punctured with only one attempt in the present study (89.3%) was much higher than that of the previous randomized trial (52.1%) (14). As a result, in the present study, patients were able to minimize the trauma of the radial artery due to puncture. Second, in the DRA, since mechanical stimulation by vessel cannulation to the proximal radial artery can be avoided, even if the vessel diameter is relatively small compared to the proximal radial artery, the rate of RAS is low, and treatment with NTG alone is considered to have a sufficient spasmolytic effect. Third, a hydrophilic, thin-wall radial sheath was used in all 400 patients undergoing CAG or PCI in our study, it is considered that mechanical stimulation by sheath cannulation was relatively less than previous studies.

Previous studies regarding the combination of various drugs including nitroglycerin and calcium channel blockers (CCBs) are summarized in Supplementary Table 3. Various spasmolytic solutions have been proposed and are widely used, including a spasmolytic solution mixed with NTG and verapamil drugs first introduced by Kiemeneji et al. (18). This spasmolytic solution is effective in preventing RAS because CCBs, such as verapamil and diltiazem, reduces the influx of calcium into vascular and arterial smooth muscle cells resulting in additional vasodilation effects (19), while NTG relax smooth muscle resulting in vasodilation. The spasmolytic effects of CCB and nitrate agents have a variable effect on RAS. Previous VITRIOL trial showed the preventive use of verapamil 5 mg offers no advantage over *ad hoc* application in terms of access site conversion rates and RAS (20). Dharma et al. reported that CCB (diltiazem) showed no advantage compared to NTG alone in the prevention of RAS in PRA. In addition, although commonly used, verapamil carries serious adverse effects including persistent hypotension, bradycardia, or ventricular asystole particularly in patients with decreased left ventricular function or conduction disturbances (9). Therefore, if verapamil is used in coronary catheterization via the DRA, it is important to monitor patients for these adverse effects. In the current study, a transient drop in the diastolic blood pressure without changes in the heart rate was observed in patients treated with additional verapamil compared to those treated with NTG alone. The results of our study suggest that only the use of NTG via intra-arterial sheath is sufficient to prevent RAS without serious side effect in patients scheduled for CAG or PCI via the DRA.

Some limitations of our study should be noted. First, the trial was conducted in a single, high-volume institution by experienced operators. Our results may not be applicable to lower-volume centers or to operators with less experience of DRA. Furthermore, our results should be carefully interpreted because V-SPA study was conducted with the concept of exploratory trial without enough statistical power. Therefore, multicenter studies are warranted to confirm the findings of the present study. Second, the current study excluded patients with hypotension, marked bradycardia, conduction disturbance or severely depressed left ventricular systolic function. Caution should be applied when generalizing these results to patients who do not meet the including criteria used in this study. Third, cardiac catheterization with 4- or 5-Fr thin-wall sheath was performed in 91.0% of the study population during the use of spasmolytic agents. In addition, we excluded acute coronary syndrome including acute myocardial infarction. Thus, further studies should be conducted in patients undergoing urgent PCI via the DRA using a large-bore sheath directly after successful puncture to investigate treatment regimens for preventing RAS. Fourth, we did not perform a repeated radial angiography to evaluate catheter induced spasm after coronary procedure. Fifth, in the current study, the dose of verapamil used was 2 mg, not 2.5 or 5 mg. However, there are no standard regimen of spasmolytic regimen in recent published randomized studies regarding the distal radial approach (13, 14, 21, 22). Therefore, our study finding could suggest standard spasmolytic regimen for DRA.

## Conclusion

In this randomized trial, intra-arterial injection of only NTG is sufficient for the prevention of RAS during CAG or PCI via the DRA compared with a cocktail regimen of NTG plus verapamil. Moreover, NTG plus verapamil is associated with a significant decrease in diastolic BP compared with usage of only NTG.

## Data availability statement

The raw data supporting the conclusions of this article will be made available by the authors, without undue reservation.

## Ethics statement

The studies involving human participants were reviewed and approved by Institutional Review Board of Yongin Severance Hospital. The patients/participants provided their written informed consent to participate in this study.

## Author contributions

O-HL and YK: study concept and design and drafting for the manuscript. O-HL, JR, YK, JC, DJ, EI, and D-KC: acquired the data. YK and D-KC: supervised the progress of the study. O-HL, JR, YK, and N-HS: acquisition, analysis, and interpretation of data. O-HL, JR, YK, N-HS, EI, D-KC, and DC: critical revision of the manuscript for important intellectual content. All authors listed have made a substantial, direct, and intellectual contribution to the work, and approved it for publication.
