# Supplementary material for: Comparison of spasmolytic regimen for prevention of radial artery spasm during the distal radial approach: A single-center, randomized study
Source: Front Cardiovasc Med. 2023 Mar 1;10:1007147. doi: 10.3389/fcvm.2023.1007147 (PMC10014463; doi:10.3389/fcvm.2023.1007147)

**SUPPLEMENTAL MATERIAL to**

**Comparison of Spasmolytic Regimen for Prevention of Radial Artery Spasm During the Distal Radial Approach: A Single-Center, Randomized Study**

Oh-Hyun Lee, MD, Ji Woong Roh, MD, PhD, Yongcheol Kim, MD, PhD, Nak-Hoon Son, PhD, Jay Yi Cho, RT, Daeseok Jang, RT, Eui Im, MD, Deok-Kyu Cho, MD,
Donghoon Choi, MD, PhD

**CONTENTS**

**Table S1. Differences in radial artery diameter before and after usage of spasmolytic agents 2**

Table S2. Summary of patients with severe radial artery spasm 3

Table S3. Summary of previous studies regarding the cocktail solution for prevention of radial artery spasm 4

Figure S1. Representative case of measurement of radial artery diameter 6

**Table S1. Differences in the radial artery diameter before and after using spasmolytic agents**

| Variables | NTG group (n=200 ) | | | Cocktail group (n=200 ) | | |
| --- | --- | --- | --- | --- | --- | --- |
|  | Before injection of spasmolytic agents | After injection  of spasmolytic agents | *P* value | Before injection of spasmolytic agents | After injection  of spasmolytic agents | *P* value |
| Most spastic area |  |  |  |  |  |  |
| Radial artery diameter | 1.83±0.35 | 2.22±0.28 | < 0.01 | 1.79±0.33 | 2.14±0.23 | < 0.01 |
| Reference area |  |  |  |  |  |  |
| Radial artery diameter | 2.66±0.16 | 2.90±0.21 | <0.01 | 2.65±0.13 | 2.89±0.15 | < 0.01 |

Data are presented as the mean ± SD.

**Table S2. Summary of patients with severe radial artery spasm**

| No. | Gender | Age | Group | dRA | Puncture time, sec | Sheath/Catheter  size, Fr | Catheter  number | PCI | Total  Procedure time, min | Clinical Vasospasm, Grade | Treatment with vasodilating agent |
| --- | --- | --- | --- | --- | --- | --- | --- | --- | --- | --- | --- |
| 1 | Woman | 71 | NTG | Left | 56 | 5 | 2 | No | 15 | 2 | 1 |
| 2 | Woman | 65 | Cocktail | Left | 160 | 5 | 2 | No | 13 | 2 | 1 |
| 3 | Men | 35 | NTG | Left | 326 | 5 | 2 | No | 18 | 2 | 1 |
| 4 | Men | 66 | Cocktail | Left | 56 | 5 | 2 | No | 13 | 2 | 0 |
| 5 | Men | 66 | NTG | Left | 150 | 7 | 3 | Yes | 106 | 2 | 1 |
| 6 | Men | 61 | Cocktail | Left | 47 | 5 | 2 | No | 13 | 2 | 1 |
| 7 | Men | 55 | Cocktail | Left | 220 | 7 | 4 | Yes | 56 | 2 | 1 |

Abbreviation: DRA, distal radial access; BP, blood pressure.

**Table S3. Summary of previous studies regarding the cocktail solution for prevention of radial artery spasm.**

| Study | Radial cocktail solution | Results |
| --- | --- | --- |
| Kiemeneij, et al. (2003)^1^ | Verapamil 5mg plus nitroglycerin 200ug vs. placebo | Incidence of RAS is high in placebo group (14% vs. 34%) |
| Rouiz-Salmeron, et al. (2005)^27^ | Phentolamine 2.5mg vs. verapamil 2.5mg | RAS rate was low with verapamil compared with phentolamine  (Phentolamine vs. verapamil 23.2% vs. 13.2%) |
| Varenne, et al. (2006)^3^ | Placebo vs. Verapamil 2.5mg  vs. Verapamil 5mg vs. molsidomine 1mg vs. verapamil 2.5mg plus molsidome 1mg | RAS rate is lowest by combination of verapamil and molsidomine  (Placebo, 22.2% vs. Verapamil 2.5mg, 8.3% vs. Verapamil 5mg, 7.9% vs. molsidomine 1mg, 13.3% vs. verapamil 2.5mg plus molsidome 1mg, 4.9%) |
| Chen, et al. (2006)^4^ | Nitroglycerin 100 ug plus UFH plus verapamil 1.25mg (Group A) vs. UFH plus nitroglycerin 100 ug (Group B) vs. UFH (Group C) | Strong statistical difference between groups A and C and Groups B and C. No statistically significant difference among group A and B.  (Group A vs. Group B vs. Group C, 3.8% vs. 4.4% vs. 20.4%) |
| Kim, et al. (2007)^5^ | Nicorandil 4mg vs. verapamil 200 ug | RAS rate is same in both group (50.7% vs. 52.0%) |
| Dharma S, et al. (2012)^6^ | Nitroglycerin 200 ug vs. diltiazem 2.5mg plus nitroglycerin 200 ug | RAS rate is same in both groups (7% vs. 5%) |
| Hizoh, et al. (2014)^7^ | Verapamil 5mg vs. placebo | Incidence of RAS is similar in both groups (1.7% vs. 1.0%) |
|  |  |  |
|  |  |  |
|  |  |  |

Abbreviation: DRA, distal radial access; BP, blood pressure.

1. Kiemeneij F, Vajifdar BU, Eccleshall SC, Laarman G, Slagboom T, Wieken Rvd. Evaluation of a spasmolytic cocktail to prevent radial artery spasm during coronary procedures. *Catheter Cardiovasc Interv.* 2003;58:281-284.

2. Ruiz‐Salmerón RJ, Mora R, Masotti M, Betriu A. Assessment of the efficacy of phentolamine to prevent radial artery spasm during cardiac catheterization procedures: a randomized study comparing phentolamine vs. verapamil. *Catheter Cardiovasc Interv.* 2005;66:192-198.

3. Varenne O, Jégou A, Cohen R, et al. Prevention of arterial spasm during percutaneous coronary interventions through radial artery: the SPASM study. *Catheter Cardiovasc Interv.* 2006;68:231-235.

4. Chen C-W, Lin C-L, Lin T-K, Lin C-D. A simple and effective regimen for prevention of radial artery spasm during coronary catheterization. *Cardiology*. 2006;105:43-47.

5. Kim SH, Kim EJ, Cheon WS, et al. Comparative study of nicorandil and a spasmolytic cocktail in preventing radial artery spasm during transradial coronary angiography. *Int J Cardiol.* 2007;120:325-330.

6. Dharma S, Shah S, Radadiya R, Vyas C, Pancholy S, Patel T. Nitroglycerin plus diltiazem versus nitroglycerin alone for spasm prophylaxis with transradial approach. *J Invasive Cardiol*. 2012;24:122-5.

7. Hizoh I, Majoros Z, Major L, et al. Need for prophylactic application of verapamil in transradial coronary procedures: A randomized trial: The vitriol (is verapamil in transradial interventions omittable?) trial. *J Am Heart Assoc*. 2014;3:e000588.

**Figure S1. Representative case of measurement of the radial artery diameter**


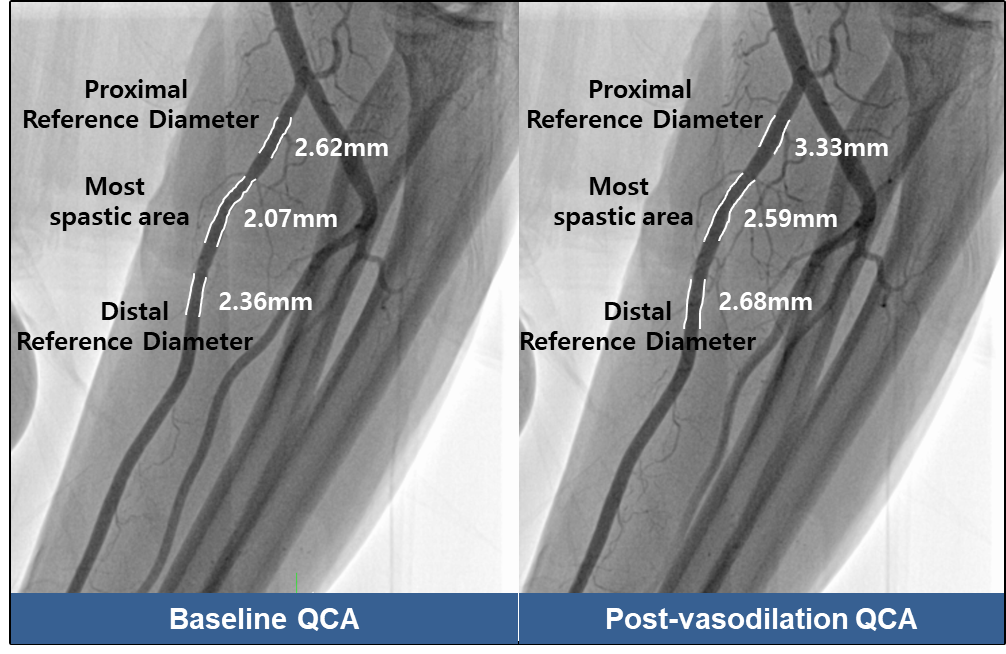

Supplement: Supplementary file 1 [file Data_Sheet_1.docx]
